# Modulation of Habitat-Based Conservation Plans by Fishery Opportunity Costs: A New Caledonia Case Study Using Fine-Scale Catch Data

**DOI:** 10.1371/journal.pone.0097409

**Published:** 2014-05-16

**Authors:** Marilyn Deas, Serge Andréfouët, Marc Léopold, Nicolas Guillemot

**Affiliations:** 1 UR-CoRéUs, Institut de Recherche pour le Développement, Laboratoire d'Excellence, CORAIL, Nouméa, New Caledonia; 2 UR-CoRéUs, Institut de Recherche pour le Développement, Laboratoire d'Excellence, CORAIL, Port-Vila, Vanuatu; 3 Fisheries Department of Vanuatu, Port-Vila, Vanuatu; 4 Nicolas Guillemot Consultant, Noumea, New Caledonia; University of Waikato (National Institute of Water and Atmospheric Research), New Zealand

## Abstract

Numerous threats impact coral reefs and conservation actions are urgently needed. Fast production of marine habitat maps promotes the use of habitat-only conservation plans, where a given percentage of the area of each habitat is set as conservation objectives. However, marine reserves can impact access to fishing grounds and generate opportunity costs for fishers that need to be minimized. In New Caledonia (Southwest Pacific), we used fine-scale fishery catch maps to define nineteen opportunity costs layers (expressed as biomass catch loss) considering i) total catches, ii) target fish families, iii) local marine tenure, and iv) gear type. The expected lower impacts on fishery catch when using the different cost constraints were ranked according to effectiveness in decreasing the costs generated by the habitat-only scenarios. The exercise was done for two habitat maps with different thematic richness. In most cases, habitat conservation objectives remained achievable, but effectiveness varied widely between scenarios and between habitat maps. The results provide practical guidelines for coral reef conservation and management. Habitat-only scenarios can be used to initiate conservation projects with stakeholders but the costs induced by such scenarios can be lowered by up to 50–60% when detailed exhaustive fishery data are used. When using partial data, the gain would be only in the 15–25% range. The best compromises are achieved when using local data.

## Introduction

In designing reserve networks to meet international, national and local targets, planners typically set specific goals for habitat conservation, with targets for a given percentage of the total area of each habitat found inside the focal domain. When such given percentage of the total area of each habitat completely defines the conservation objectives, without any other objectives, this is what we call a habitat-based conservation plan. This type of plan is attractive because it should theoretically include all the biological species and functional groups found in these habitats, whether these species and groups are known or not [1]. These plans are also straightforward to compute once habitat maps are available, which is routine task with remote sensing technology [2]. As a consequence, these habitat-only designs are not data demanding, reserve networks can be easily produced, and as such they can be extremely useful to initiate discussions with the various stakeholders with a first exploratory plan in hand. The proposed plans can be enhanced iteratively when new data become available and when new objectives are defined [3].

However, such plans are idealistic in the sense that they ignore local socio-economic constraints [4], [5], [6], [7], [8]. There is a wide consensus that when avoiding socio-economic realities, conservation actions are likely doomed, with poor compliance if conservation brings significant pressure on local community livelihoods [9]. In the marine realm, a habitat-only design brings penalties, and costs, to fishers. It is therefore needed to evaluate the socio-economic impact when using a simple (in terms of required data) habitat-based conservation design and compare with marine reserve scenarios that minimize costs. Better understanding of how socio-economic constraints modulate habitat-based conservation plans would help managers knowing their limits and building confidence in using such plans. This is especially true in coral reef environments where spatially-explicit socio-economic data are often lacking and conservation actions are urgent. Indeed, better conservation measures are widely needed for many reefs worldwide, at risk from coastal development, unsustainable resource use and climate change [10]. Therefore, we here aim to test in a coral reef environment if habitat-based plans are robust to socio-economic constraints and how valid they would be when socio-economic data become available. Specifically, we test how fishery opportunity costs modulate habitat-based plans.

Opportunity costs are defined as the costs of foregone opportunities; in other words, they are a measure of what could have been gained via the next-best use of a resource had it not been put to the current use [11]. They are increasingly used to account for the socio-economic costs of conservation, especially for fisheries [12]. Opportunity costs for fisheries have been previously considered in coral reef environment: Weeks et al. [13] have used socio-economic proxies to infer opportunity costs in Philippines and Adams et al. [14] have estimated opportunity costs in Fiji both from fishing catch per unit effort surveys and from what could be the production of a reef using underwater fish visual census. Availability of fine-scale socio-economic data are expected to better optimize the costs of conservation [4], particularly where local tenure or resource use rights over marine spaces covers small areas, a common feature of many countries in the Coral Triangle and Southwest Pacific. Otherwise, for coral reefs, fishery data have also been used to assess trade-offs between conservation and fishers activities, but no opportunity costs were estimated or mapped [7]. This gap is explained by the difficulty, or lack of resources, devoted to achieving a precise mapping of the fishing effort and catch in coral reef environment. In fact, this information is sorely missing for virtually all reefs of the planet that are under stress due to fishing activity [14], [15]. Data are more frequently available for temperate commercial fisheries that are well monitored using observers and logbooks [12]. Most previous studies that have collected spatially-explicit information on coral reef fisheries have only included locations of fishing grounds, sometimes detailed by targeted species and gear types [7], [16], [17], [18], [19], [20].

Use of fine-scale maps of fishery catch to directly infer opportunity costs would be a substantial improvement over use of models or proxies of socio-economic costs. Recently, Guillemot and Léopold [21] released a thorough compilation of coral reef fishery activities for one region of New Caledonia, in the Voh-Koné-Pouembout area. The result is a fishery atlas with fine scale maps of total production (or catch), and detailed catch by key fish family, gear type and tenure. To the author's knowledge, no similar data set has been used in any given coral reef locations to analyze conflicts between conservation and fishery activities. The associated database is a gold mine of spatially explicit information that we used together with different types of habitat maps to answer the following questions:

- How do fishery opportunity costs modulate habitat-based marine reserve plans in a coral reef environment?
- How does the thematic richness of the habitat maps influence the results?
- How does the type of opportunity costs (fish family-related, location-related, gear-related) influence the results, and do these different types provide redundant information? Conversely, can data from one location be used to efficiently make decisions for another location?
- What are the practical lessons for coral reef managers in charge of implementing conservation actions in heavily fished areas?

To answer these questions, we compare habitat-based only conservation plans with habitat-based conservation plans constrained by opportunity costs. This quantitative comparative analysis was made possible due to the unique availability of the Voh-Koné-Pouembout fishery atlas, and two types of habitat maps.

## Materials and Methods

### Ethics statement

This work did not involve manipulations of animals, thus it did not involve endangered or protected species. Modeling methods did not require specific permissions from any relevant body as they are harmless and meet all applicable standards for the ethics of experimentation and research integrity.

### Study site

**Figure 1:**
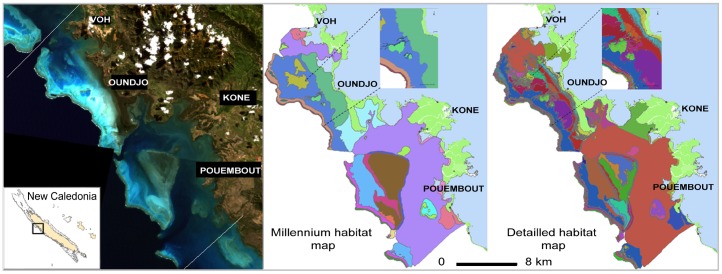
View and maps of the study area. Left panel: view of the Voh-Koné-Pouembout coral reefs and lagoons. The white lines show the extent of the study area. Right and Central panels: habitat maps used for this study, Millennium habitat map and Detailed habitat map respectively. Legends are not shown here given their length (23 and 106 classes) but the differences in levels of details are visible.

Voh Koné Pouembout (VKP) is a rural area of the north-west coast of New Caledonia. The barrier reef system, at a distance of 6 km on average from the coast, includes four passes that delimitate the 211 km^2^ study area (Fig. 1). Nickel mining is a main driver of the New Caledonia economy [22]. A nickel mining project in the VKP region recently boosted the local economy leading to urban development and a population increase of 18% between 2004 and 2009. Around 6400 newcomers are expected to settle by 2015 [23], [24] and to increase the demand for fishery resources. This was the reason why fishery data were collected in 2007, and compiled in a comprehensive atlas of fishery maps, to serve as a future baseline [15].

### Habitat maps

The lagoon and reefs include a broad range of habitats. To evaluate the possible influence of habitat map thematic resolution and complexity on the results, our study design included two distinct habitat maps. The first map included 23 different habitats, defined by their geomorphological attributes as listed by the Millennium Coral Reef Mapping Project [25]. The interest of using this map is that similar products are available for many countries and regions worldwide [26], [27], and lessons learned with this product can provide guidance elsewhere. The second map, much more complex, included 106 habitats defined by both geomorphological and benthic cover attributes (substrate, percentage cover, and architecture of numerous coral, algae and seagrass communities). This map was created following a user approach [26], with a Quickbird satellite image at 2.4 meter spatial resolution. Figure 1 illustrates the differences between these two maps. Geographical Information Systems (GIS) shapefiles of both products are available on request to the corresponding author.

### VKP reef fishery data

**Figure 2:**
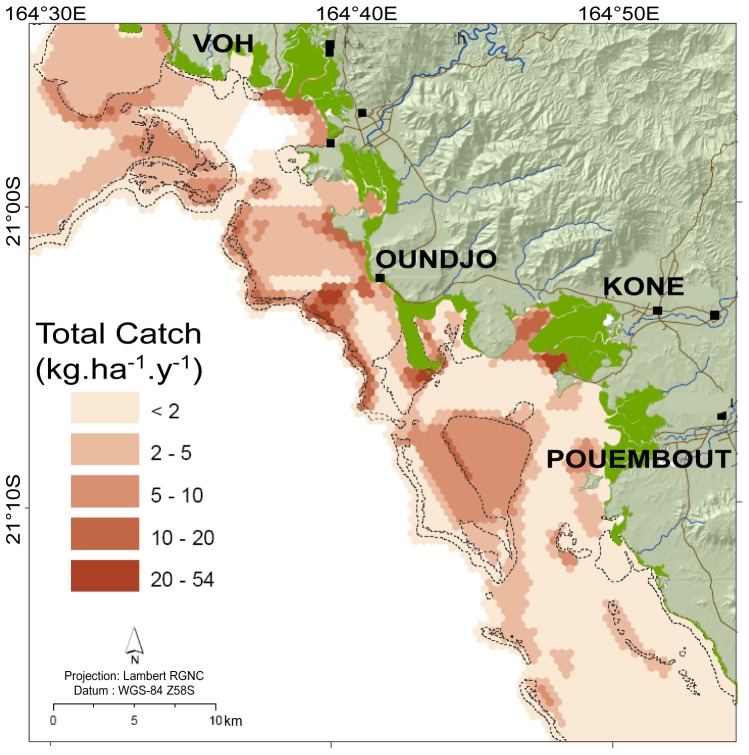
Map of *Total Catch* for the Voh-Koné-Pouembout area. Example data included in the fishery atlas used to compute opportunity costs (From Guillemot and Léopold [21]).

Methods for the production of the fishery atlas are detailed in Léopold et al. [15]. These authors describe four different mangrove and coral reef fisheries targeting invertebrates or finfish around New Caledonia. The fisheries at these sites were mapped following a five-step framework: 1) stratified random sampling of regular fishers; 2) collection of fishers' knowledge on fishing areas, fishing effort, and catch characteristics through map-based interviews; 3) data integration into a spatial geodatabase; 4) statistical extrapolation of fisher data to the fishery scale; and 5) mapping of catch, effort, and catch per unit of effort (CPUE) for each fishery. An example of product is provided Figure 2. Fishery maps are representations of catch data and are therefore also shaped by indirect factors affecting fishing locations, such as clan disputes, customary reserves and taboos, sea conditions, or fishers' personal preferences. Furthermore, catch maps indirectly reflect to some extent the preference habitat distribution and home range of each target species over a one-year period.

Most regular fishers in the area were present in two small multicultural and multi-ethnic small towns (Koné and Pouembout) and one Melanesian village (Oundjo) that totaled about 310 fishing boats. Fishing practices differed between these localities and communities. In Pouembout and Koné, fishing was mostly a recreational week-end activity, involving few fishers using large boats and selective gears like spear gun and handline. Annual fishing effort catches and mean yield were consequently relatively low. Conversely, Oundjo fishers used highly efficient but non-selective gears like gillnets, with small boats during the week, leading to high fishing yield and annual fishing effort. About 30% of catches were unofficially sold directly to local markets. The activity was thus driven by both income and food needs, resulting in more intense exploitation than subsistence fishing only [28].

Socioeconomic and cultural differences between fishers explained the spatial partitioning of the lagoon fishing area. Noteworthy is the Oundjo's customary exclusive fishing area, a 61 km^2^ restricted access area closed to outsiders. Oundjo fishers are also active outside this area but since they used small boats, they favored shore-fishing on inner and fringing reefs near their village. Fishers from Koné and Pouembout consequently use only the southern part of the lagoon area, and travel further from the coast, including to the barrier reef [23].

Overall fishing pressure was quite low in 2007, with an estimated average fish catch of 0.24 t.km^−^^2^.year^−1^. However, in restricted sites of the barrier reef, mangroves and fringing reef areas inside Oundjo's exclusive area, fishing pressure reached 5 t.km^−^^2^.year^−1^, which was considered as a threshold for sustainable reef fisheries [29].

### Reef fishery opportunity costs

Estimated annual mean yield, total effort, total catch, catch by gear type, catch by locality and catch by fish family were mapped by Guillemot and Léopold throughout the study area using a hexagonal grid [21]. Each of these data layers offers the possibility to run a different scenario, and directly compute an opportunity cost for each grid cell. For this; we assumed that if a grid cell is turned into a no take area, the associated catch loss (total, by locality, by gear type, by fish family) for fishers represents the opportunity cost.

From the fishery atlas, we considered 19 catch data layers to define opportunity costs and conservation scenarios. These are (Table 1):

**Table 1:** Opportunity costs and their related data layers, used to define the different scenarios, identified by indexes.

General	Data and Cost index	Catches by locality	Data and Cost index	Catches by gear	Data and Cost index	Catches by fish family	Data and Cost index
Total catches	1	Koné	2	Gillnet	5	Lethrinidae	8
		Oundjo	3	Spear gun	6	Acanthuridae	9
		Pouembout	4	Hand line	7	Mugilidae	10
						Scaridae	11
						Serranidae	12
						Siganidae	13
						Lutjanidae	14
						Haemulidae	15
						Carangidae	16
						Sparidae	17
						Gerreidae	18
						Kyphosidae	19

1. total catches,
2. catches by locality, to compare the effectiveness of conservation plans designed according to local fishery data with plans designed according to fishery data from another area (or for the entire area),
3. catches by gear, to compare the effectiveness of conservation plans designed according to a sub-set of fishermen with plans designed according to the entire fishing population,
4. catches by fish family, to compare the effectiveness of conservation plans designed using limited set of data with plans designed using an exhaustive list of target fish families and total catch.

### Conservation designs

Planning units were defined by a grid of hexagons (side length = 500 m, surface of 21.6ha) mimicking the grid used for the fishery maps (Fig. 2). The number of planning unit was 1525 and 1531 respectively for the Millennium and detailed map.

Our conservation objective was to represent at least 20% of all mapped habitats in the area. This threshold was used as a compromise between the Convention on Biological Diversity 11^th^ target of 10% established in 2010 and the 20 to 30% target more likely needed in the longer term for effective conservation in the Pacific [7]. First, a “habitat-only plan” was computed, to define a habitat-based reserve network without using any cost constraint. Then, we included the constraint to minimize opportunity costs for each of the 19 cost layers, corresponding to 19 cost scenarios. Each cost was used independently, and no combination of costs was used.

The Marxan software was used to compute the networks,. Marxan is a freely available conservation planning software based on simulated annealing. It solves a minimum-set problem where the objective is to minimize the cost of a reserve network while ensuring conservation feature targets are met [30]. We searched for an optimal boundary length modifier (BLM) following Stewart and Possingham [31]. This parameter controls the trade-offs between minimizing costs and allowing for a more compact and realistic reserve. The optimal BLM was in the same range, 0.0025, for the habitat and the *Total Catch* cost scenarios, and we applied the same BLM value for all scenarios. Each scenario was run for both habitat maps. Eventually, this resulted in forty different reserve networks (twenty designs for each map, including one habitat-only design, and 19 scenarios with cost constraints).

### Scenario evaluation and comparison

Efficient cost scenarios minimize opportunity costs while meeting all conservation targets. In other words, an efficient scenario minimizes fishery catch loss while meeting the 20% habitat representation target for each habitat. First, we studied the effect of each scenario on the matching cost, which means how the inclusion of a cost as a constraint in the design influenced its own production. For instance we looked at how using a cost constraint on “Lethrinidae” influences the Lethrinidae catch that is locked in the reserves. Second, we studied the effects of each scenario on the total catch loss. For instance we looked at how using the cost constraint on “Lethrinidae” influences the total catch that is locked in the reserves. Indeed, total catch is a global representation of the potential production on the area, without distinction between fish families, gears or groups of fishermen, and this level of catch was used here as a reference.

To achieve these objectives, we described the reserve networks according to:

1. conservation targets met (at least 20% of each habitat included in the network). Specifically, the variable *Mv* from Marxan represents the achieved percentage of habitat conservation target. *Mv* can range from 0 (habitat not included in the design) to 1 (conservation target fully satisfied). The *Mv*>0.95 threshold marks the limit where the conservation target was considered satisfactory. We then counted how many targets (*N*) were not met to rank the scenarios.
2. *P*_i_, as the initial catch for data layer *i* (*i* = 1 to 19, Table 1).
3. *h,* as the remaining catch when using the habitat-only scenario, and *H* = *P-h* as the resulting opportunity cost of the habitat-only scenario. *H* (*H* for *H*abitat) can be expressed for Total Catches (*H_1_*) but also for any of the other 18 other data layers (*H_2_* to *H_19_*) that can be used to define opportunity costs.
4. *c*, as the remaining catch for the same data layer *i* that was used to define opportunity costs, and *C = P_i_-c_i_*, as the resulting opportunity cost. Catches *C* (*C* for *C*ost layer) can be computed for each of the 19 selected data layers. For instance, the variable *C_4_* will quantify how the scenario based on *Oundjo* cost (scenario 4 in Table 1) reduces *Oundjo* catch loss compared to the habitat-only scenario *H_4_*.
5. *t*, as the total remaining catch in a scenario with opportunity cost and *T = P_1_-t* as the resulting opportunity cost. *T* can also be computed for each of the 19 selected data layers. For instance, the variable *T_8_* will quantify how the scenario based on *Lethrinidae* cost (scenario 8 in Table 1) reduces total catches loss compared to the habitat-only scenario *H_1_*. Note that *T_1_* = *C_1_*.

All metrics above (*P, H, T, C*) are expressed in kg.ha-^1^.yr^−1^ and are computed for the entire area. Table 2 summarizes these variables.

**Table 2:** Variables used to describe the different scenarios.

Descriptor	Meaning of descriptor
*N*	Number of habitats with *Mv* <0.95 (*i.e*., conservation objective not met)
i ∈[1]; [19]	Index of cost layer (cf Table 1)
*P_i_*	Initial production available for data layer *i*
*H_i_*	Value of opportunity cost for data layer *i* in the habitat-only design
*C_i_*	Value of opportunity cost for data layer *i* with the corresponding cost *i* scenarios
*T_i_*	Value of opportunity cost for Total Catch, for the cost *i* scenario (*T_1_* = *C_1_*)

More metrics are theoretically available. In particular, we could cross-analyze each scenario and cost, for instance by looking at the influence of the *Lethrinidae* cost scenario on *Scaridae* opportunity cost. For simplicity sake, we did not present here these results, as the main trends and results can be described from the above metrics.

To evaluate and compare the effectiveness of the different scenarios, we used different indices summarized in Table 3:

**Table 3:** Selected metrics of scenario effectiveness, privileging *Total Catch* (*i* = 1) for reference.

Variable	Description
%*H_i_* = *H_i_*/*P_i_*	Ratio opportunity cost *vs* initial catch of data layer *i* (*i* = 1 to 19, cf. Table 1), for the habitat only design
*%C_i_ = C_i_/P_i_*	Ratio opportunity cost *vs* initial catch of data layer *i* (*i* = 1 to 19, cf. Table 1), for the opportunity cost *i* scenario
%*T_i_* = *T_i_*/*P_1_*	Ratio opportunity cost of Total Catch *vs* initial Total Catch, for the opportunity cost *i* scenario
*E_i_* = 1-(*C_i_*/*H_i_)*	Effectiveness of cost *i* scenario considering opportunity cost i (*100 if given in %).
*E_1,i_*_ = _1-*(T_i_*/*H_1_)*	Effectiveness of cost *i* scenario considering total catches opportunity cost (*100 if given in %)

- First, *%H* expresses the percentage of potential annual catch unavailable to fishers based on the habitat only scenario, or, in other words, the initial production “locked-in” by the habitat-only scenario. A *%H_i_* can be computed for any of the 19 possible scenarios *i*. The higher *%H_i_*, the more costly is the habitat-based design for fishers that yielded the production *P_i_*.
- Second, *%C* expresses the percentage of potential annual catch unavailable to fishers based on the corresponding cost scenario. A *%C_i_* can be computed for any of the 19 possible scenarios *i*. The higher *%C_i_*, the more costly is the scenario for fishers that yielded the production *P_i_*.
- Third, *%T* expresses the percentage of *Total Catch* locked in, for any considered scenario. Among the 19 different possibilities in selecting a reference, we favored the comparison of the different cost scenarios relatively to the *Total Catch* information (*i* = 1). A *%T_i_* can be computed for any of the 19 possible scenarios *i*. The higher *%T_i_*, the more costly is the scenario for fishers that yielded the production *P_1_*.

Finally, we define the effectiveness *E* of a scenario as the reduction of opportunity cost between a cost-based scenario and the habitat-only, scenario, with *E* = 1-*C/H* (Table 3). Then, the effectiveness *E_1_* is similar to *E* but considering *Total Caches* as the reference, or *E_1_* = 1-*T/H_1_* (Table 3). These metrics provide different references (catch for scenario *i* or *Total Catch*) on the percentage of opportunity costs induced by the habitat-only scenario that have been effectively reduced when using fishery cost constraints. *E* and *E_1_* ϵ [0,1] and are unitless (or expressed as percentage). The higher the *E* or *E_1_* value, the more efficient the cost scenario is compared to the habitat-based scenario. Note that *E* and *E_1_* can be negative if the cost scenario does not reduce the opportunity costs below the levels reached by the habitat-based scenario (i.e. *C*>*H* or *T*>*H_1_*). In this case, the scenario is not efficient.

**Figure 3:**
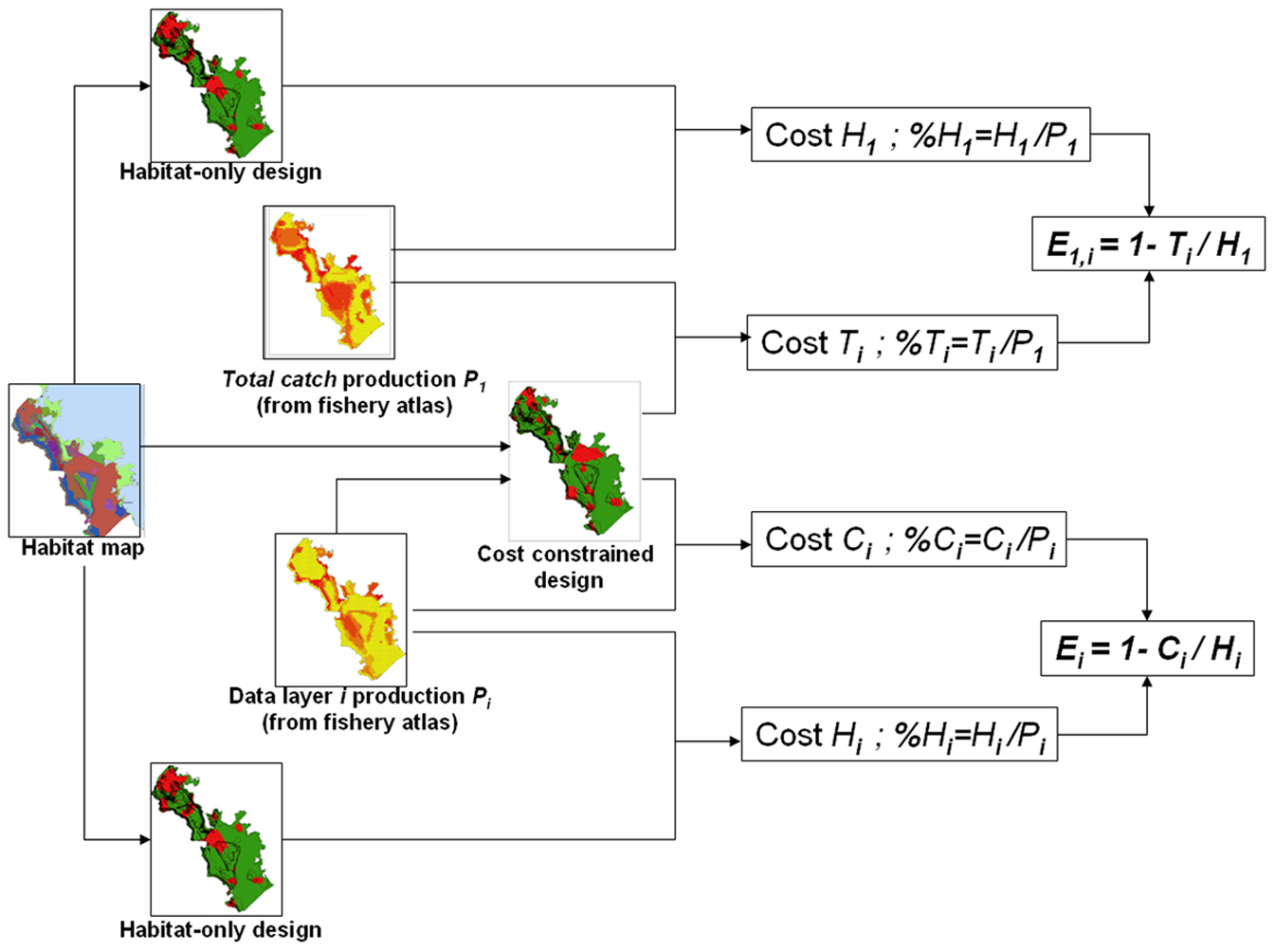
Flow chart showing how the different metrics were obtained. Habitat map and fishery Catch (or production) data are in input; then different conservation designs can be computed, with or without cost optimization. The various metrics (see text for details) can be computed and provide different outputs relative to the effectiveness of the different scenarios. The “Habitat only design” icon is repeated for clarity, to avoid crossing lines in the chart.

Figure 3 shows a flow diagram with input data, output scenarios and relationships between the different variables.

## Results

### Networks of protected areas

**Figure 4:**
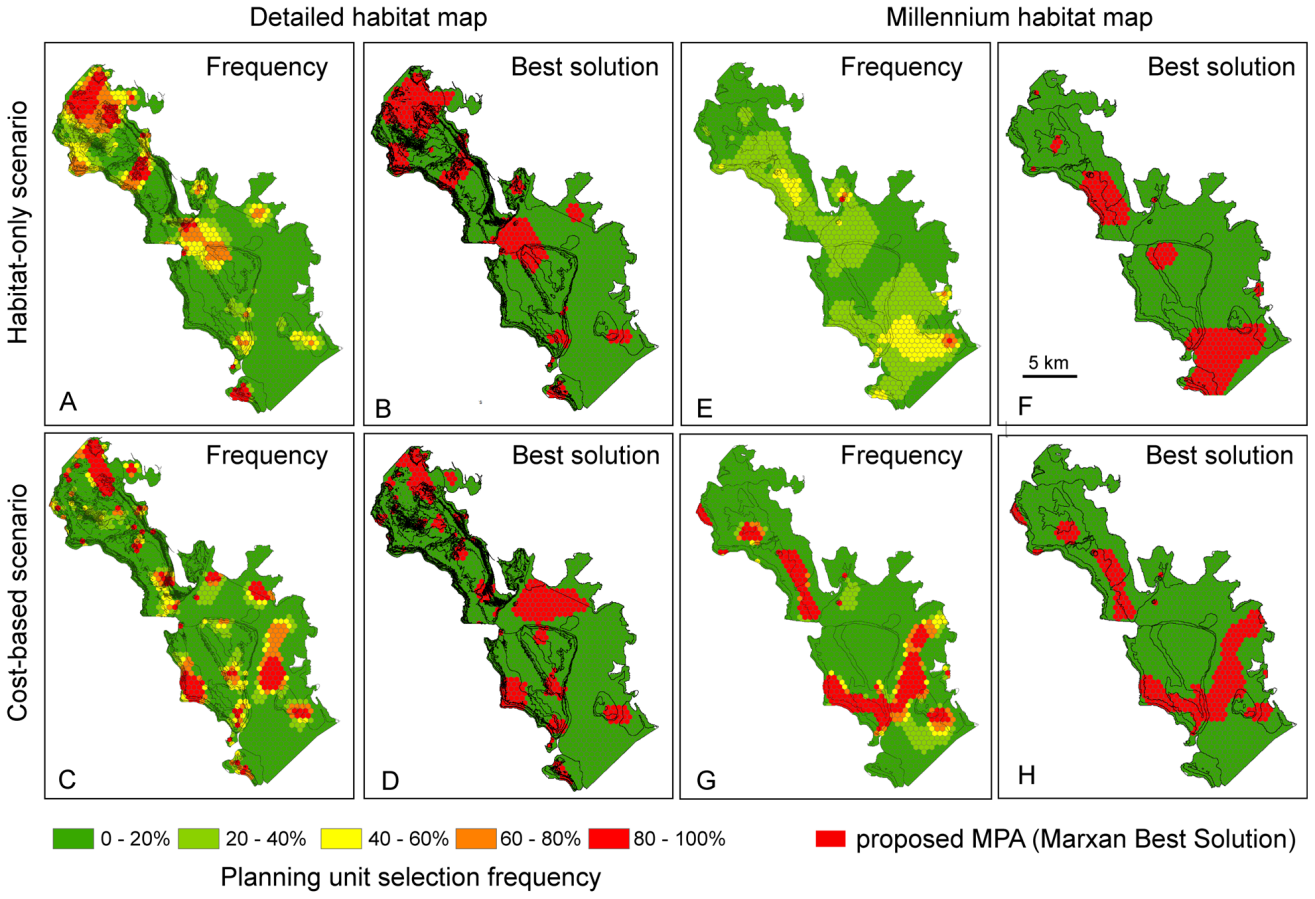
Examples of conservation designs, with best solutions given by Marxan and selection frequencies of planning units. For the two different habitat maps, the changes in proposed reserve locations are shown between a habitat-only scenario and a cost-based scenario, based here on *Total Catch* data (see Figure 2). High frequencies and best solutions are in strong agreement in some cases (panels A and B; panels G and H). When they differ in specific areas, it is because of habitats particularly abundant, such as the deep lagoon, that offer flexibility in the selection of management units (panels C and D), or because of lack of prioritary units (as shown also in low contrasted frequencies) (panels E and F).

For each design, we selected the best solution computed by Marxan as our final networks. Best solutions were consistent with planning unit selection frequencies (Fig. 4). Examples of output networks are presented in Figure 4. Figure 4 also illustrates 1) the compactness of the different reserves, due to the selection of BLM = 0.0025, 2) the differences achieved between a Millennium habitat analysis and a detailed habitat map analysis, 3) the influence of an opportunity cost constraint on a habitat-only design. In each case, this influence is high and substantially changes the distribution of the reserves in the network, as shown in Figure 4.

All metrics are provided in Tables 4 and 5. Catch values and effectiveness are provided for each scenario.

**Table 4:** Level of conservation objectives met (*N*) and lost catch (opportunity costs *H, C, T*).

			Costs *(kg.km^−^^2^.yr^−1^)*							
	*N*		*P*		*H*		*C*		*T*	
Data layer	M	D	M	D	M	D	M	D	M	D
Habitat only	1	1								
Total catches	3	7	5052	5058	736	1211	286	538	286	538
Oundjo	2	7	2552	2553	389	839	97	240	522	796
Gillnet	2	5+	2140	2141	281	550	38	162	572	883
Handline	2	**8**	1668	1672	209	361	84	156	505	816
Koné	2+	5	1411	1411	206	191	81	92	990	1613
Speargun	3	5+	1181	1181	231	284	120	153	644	888
Lethrinidae	3	6	1055	1058	158	264	79	125	511	870
Acanthuridae	1	3+	824	824	147	238	86	136	623	852
Mugilidae	**4**	4	759	759	74	156	22	77	712	1302
Scaridae	2	4	520	520	104	163	48	89	644	917
Serranidae	1	4	517	518	73	109	66	85	611	1095
Siganidae	1	2	498	498	87	120	58	99	661	1244
Pouembout	0	3	329	329	103	43	24	33	1102	1744
Lutjanidae	**4**	3	270	270	17	56	16	36	737	1252
Haemulidae	2	0	192	192	11	16	13	19	1079	1718
Carangidae	1	2	169	169	23	41	17	43	738	1676
Sparidae	1	1	100	100	3	6	4	7	1202	1753
Gerreidae	1	0	34	34	0	0	6	0	917	1915
Kyphosidae	1	4+	21	21	3	7	3	10	1259	1844

Results are provided for both maps (M for Millennium, D for the detailed map) and for all fishery data layers and scenarios, sorted and presented by decreasing *P.* In the “*N”* columns, “+” next to a number indicates that at least one habitat could not be represented at all in the design. The slight differences in *P* between the Millennium and Detailed map are due to a resolution-edge effect at the boundary of the respective map domains.

**Table 5:** Results for all metrics (*%H, %C, %T, E, E_1_*).

Data layer	*%H*		*%C*		*E (%)*		*%T*		*E_1_ (%)*	
	M	D	M	D	M	D	M	D	M	D
Habitat-only									-	-
Total catches	14.6	24	5.7	10.6	61.1	55.6	**5.7**	**10.6**	**61.1**	**55.6**
Oundjo	15.3	**32.8**	3.8	9.4	75.2	**71.4**	10.3	15.7	29.0	34.3
Gillnet	13.1	25.7	**1.8**	7.5	**86.6**	70.6	11.3	17.5	22.2	27.1
Handline	12.5	21.6	5.1	9.3	59.6	56.7	10.0	16.1	31.3	32.7
Koné	14.6	13.5	5.7	**6.5**	60.9	51.7	19.6	*31.9*	*−34.5*	*−33.2*
Speargun	19.5	24.0	10.1	12.9	48.2	46.2	12.7	17.6	12.4	26.7
Lethrinidae	15.0	24.9	7.4	11.8	50.2	52.7	10.1	17.2	30.5	28.2
Acanthuridae	17.9	28.9	10.4	16.5	41.8	42.9	12.3	16.9	15.2	29.6
Mugilidae	9.8	20.6	2.9	10.2	70.6	50.4	14.1	*25.7*	**3.3**	*−7.5*
Scaridae	**20.1**	31.4	9.2	17.1	53.9	45.5	12.7	18.1	12.5	24.3
Serranidae	14.1	21.1	12.8	16.4	**9.1**	22.3	12.1	21.6	16.9	**9.6**
Siganidae	17.5	24.1	11.7	19.9	33.2	**17.5**	13.1	*24.6*	10.1	*−2.7*
Pouembout	31.4	**13.0**	7.3	10.1	76.9	22.2	*21.8*	*34.5*	*−49.8*	*−44.0*
Lutjanidae	**6.2**	20.8	5.9	13.5	4.8	35.3	*14.6*	*24.8*	*−0.2*	*−3.4*
*Haemulidae*	5.7	8.1	6.8	10.0	*−20.9*	*−23.9*	*21.4*	*34.0*	*−46.7*	*−41.8*
*Carangidae*	13.4	24.2	10.2	25.3	23.7	*−4.8*	*14.6*	*33.1*	*−0.3*	*−38.3*
*Sparidae*	3.2	5.7	4.4	6.5	*−39.8*	*−15.4*	*23.8*	*34.7*	*−63.5*	*−44.7*
*Gerreidae*	0.2	0.6	17.0	0.6	*−10476*	7.0	*18.1*	*37.9*	*−24.6*	*−58.1*
*Kyphosidae*	14.4	34.7	14.8	47.1	*−3.4*	*−35.8*	*24.9*	*36.5*	*−71.1*	*−52.2*

Results are provided for both maps (M for Millennium, D for the detailed map) and for all fishery data layers and scenarios, sorted in the same order as in Table 4 (by decreasing *P*). Non-efficient scenarios are indicated by the data layer in italics. Best results for efficient scenarios (maximum values for *E* and *E_1_*, minimum values for other indicators) are in bold underlined, while worst results are in bold only.

### Comparison of scenarios based on conservation objectives

Table 4 presents the number of missing habitats *N* in each scenario for both habitat maps.

For the habitat-only scenario, only one habitat could not be represented for each habitat map (*N* = 1). Including costs generally led to trade-offs to satisfy conservation objectives compared to the habitat-only scenarios (i.e., *N>1*). However, the best scenarios overall (*N* = 0) were for *Pouembout*, *Haemulidae* and *Gerreidae* scenarios for the Millennium and detailed map respectively. The worst scenarios were the *Mugilidae* and *Handline* scenarios with respectively 4 and 8 missing habitats, from Millennium map and detailed map respectively.

When comparing the different values for *N* (Table 4), the detailed map plan was generally more efficient in satisfying conservation objectives than the Millennium one, considering the highest complexity and fragmentation of habitats in the detailed map. These characteristics intuitively should lead to more frequent trade-offs when trying to include 20% of the surface of all habitats.

### Comparison of scenarios based on costs

Table 4 show that for both habitat maps, almost all cost-based scenarios yielded a decreased opportunity cost (i.e., *H_i_*>*C_i_*), or in other words an increase in available biomass for fishers, when shifting from a habitat-only to a cost-based scenario. This result was expected, but the differences achieved when using the various opportunity costs appear clearly.

Noteworthy are the scenarios that surprisingly increased the opportunity costs. These were the *Haemulidae, Sparidae, Gerreidae* and *Kyphosidae* scenarios for Millennium maps, and *Carangidae* with the detailed map. These scenarios used very low catch values, and the catches appeared scattered across the maps in few habitat patches. Small changes in how these patches are included or not in the network led to high variations of effectiveness, even if the absolute costs remained low. This suggests that the effectiveness of a network constrained by low catches scattered in the seascape would not be robust to small spatial changes in the design. These scenarios are hereafter flagged as “non-efficient”.

Table 5 presents *%C, %H* and *%T* achieved for each scenario, including the non-efficient ones (in italics). For efficient scenarios *%C* is systematically lower than *%H*, as expected. The scenario that generated the lowest *%H* was *Lutjanidae* using the Millennium map (6.2%). In other words, the remaining *Lutjanidae* catch available to fishers was the least affected by the habitat-based scenario. The lowest *%C* is generated by the *Gillnet* scenario with only 1.8% of initial catch immobilized, also using the Millennium map.

Lowest *%T* is achieved for *Total Catch* scenario, for both maps (5.7% and 10.6% for the Millennium and the detailed map respectively). This implies that, to decrease the habitat-only opportunity cost, using *Total Catch* data, as the largest picture on the catch distribution, leads to better outcomes for fishers than using only a fraction of the catch information (from a sublist of species, selected catch gear or specific locality). This was also expected, but the ratio *%T* clarifies the amount of benefits for the fishers between the different scenarios. Using the *Total Catch* scenario proves to be 2 and 3 times more interesting for fishers than any other scenario.

Among the scenarios that are effective with their own corresponding costs (*E*), some are not efficient anymore compared to their effect on *Total Catch* costs (*E_1_*). This includes the *Lutjanidae, Carangidae, Koné* and *Pouembout* scenarios for the Millennium map. For instance, *Pouembout* scenario shows the second best *E* (76.9%) but also one of the worst *E_1_* (−49.8%). In other words, to decrease opportunity costs of the Millennium habitat-only design for the entire area, using only information on *Pouembout* catch is not effective at all. Similarly, the highest *E* is reached for *Gillnet* (86.6%) and *Oundjo* (71.4%) for the Millennium and the detailed map respectively. However, for *E_1_*, *Gillnet* and *Oundjo* drop to 22.2% and 34.3% respectively. Overall, on average, *E_1_* is about twice lower then *E*, with an average *E_1_* of 22.3% and 29.8% for the Millennium and detailed maps respectively, and an average *E* of 52.3 and 45.8% for the Millennium and detailed maps respectively. This means that when using a particular catch layer *i* to constrain the design, the effectiveness in decreasing the *Total Catch* loss is always lower than decreasing the corresponding catch *i* loss.

### Influence of the type of habitat map

According to *H, C*, *%H and %C* (Tables 4 and 5), using the Millennium map generates lower opportunity costs than the detailed map, for all but one (*Pouembout* scenario) of the possible type of catches. This is observed with (when using *%C*) or without (when using *%H*) the constraint of minimizing opportunity cost in the design. Therefore, all habitat maps are not equal in generating costs when using them for a habitat-only design, or for a cost-constrained design. Here, we found that using the Millennium map, less thematically rich, is less costly for fishers in all situations.

**Figure 5:**
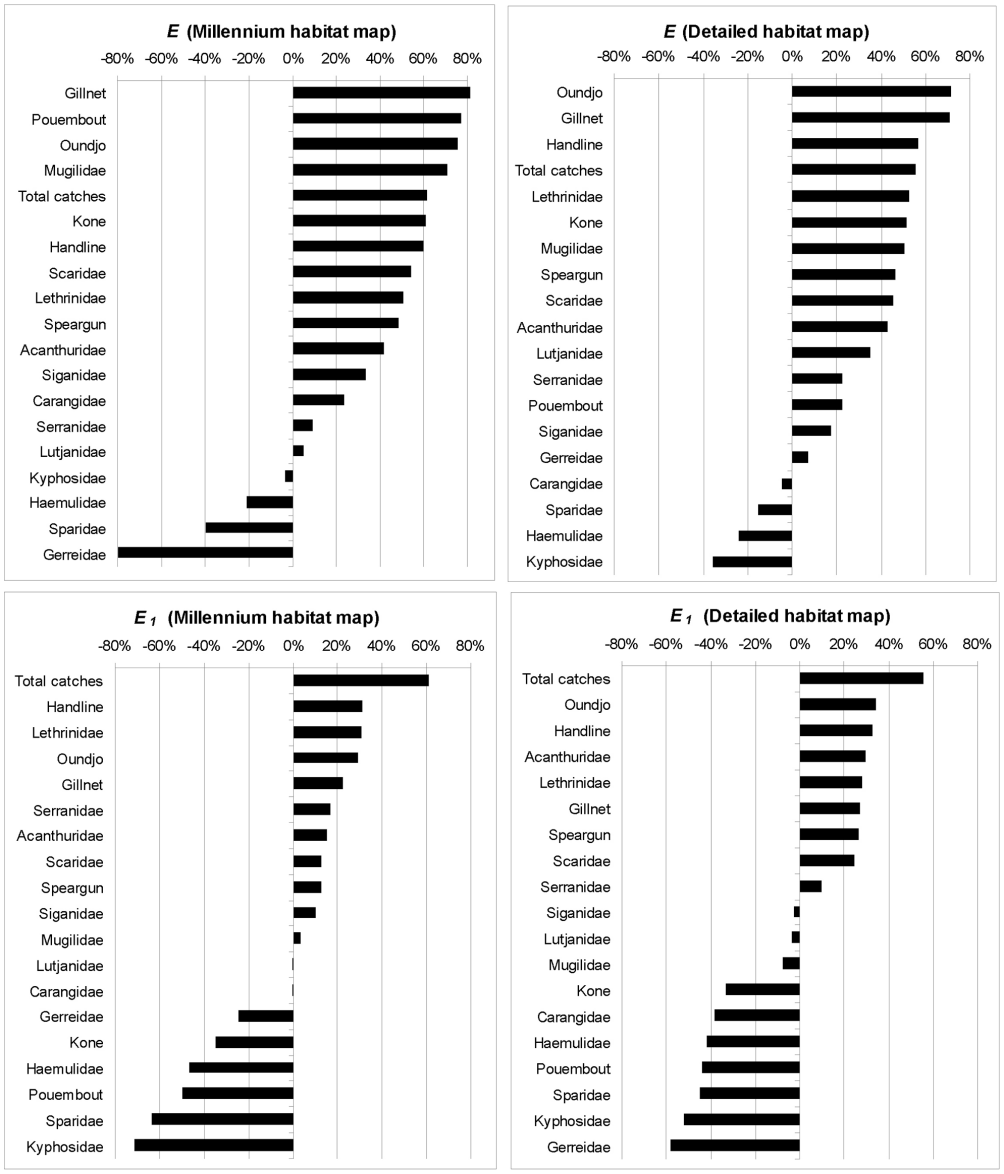
Comparison of *E* and *E_1_* achieved between the two habitat maps and the various cost-based scenarios. Negative values <−100% (see **Table 5**, *Gerreidae* family) are forced to −80%.

The pattern is slightly different with *E*, i.e. when comparing the relative benefits of a cost-based approach to decrease the opportunity costs generated by the habitat only approach (Table 5, Figure 5). *E* shows a less uniform hierarchy between map types. Specifically, the benefits of using cost constraints are higher with *Lethrinidae, Serranidae* and *Lutjanidae* when using the detailed habitat map compared to the Millennium map. The Figure 5 ranks the performances of each scenario according to *E* and *E_1_* and for each map. If a manager has the choice of the scenario and can mix at will the types of productions and habitat maps, Figure 5 suggests that Millennium map-based scenarios will offer better combination than the detailed map-based scenarios, with higher *E* values overall. But the pattern is opposite for *E_1_*. In other words, a manager would be less efficient in decreasing the loss of *Total Catch* production (due to a habitat-based scenario) with a cost-based scenario if he has to use the Millennium maps.

## Discussion

### Sensitivity of habitat-based conservation plans to fishery opportunity costs

Effective and accepted conservation will be achieved if socio-economic realities are taken into account, but conservation actions may be channeled more quickly when using easily accessible data, such as habitat maps created anew with remote sensing or from already existing archives (e.g., Millennium maps). As such, we assessed here the ability of different opportunity costs to reduce the impact of habitat-based conservation plans, using fine-scale catch data. Indirectly, we measure the cost of conservation for fishers when usng only habitat-based scenarios. In contrast with Adams et al. [14] who modeled opportunity costs using the potential production of a site, including unfished areas, we used here directly the estimated spatial catch to directly derive opportunity costs. Here, the entire focal area was fished [15].

According to our New-Caledonia case study, minimizing fishery opportunity costs modulate the habitat-only conservation plan in various ways. First, the spatial distribution of the potential protected areas changes substantially (Fig. 4), and the cost constraints generally did not inhibit the ability of reserve designs to meet the conservation objectives (20% of the surface area of each habitat) even for the most complex habitat map. Second, the potential catch loss following habitat-driven conservation can be minimized, but there are many options. The effectiveness in achieving this goal will largely depend on the type of fishery catch used and the type of habitat map as well. Our results (Tables 4, 5; Fig. 5) indicate the sensitivity of the different {fishery data, habitat map} combinations in minimizing the opportunity costs of habitat-driven scenario. The results also show that it is possible to generate costs higher than that of the habitat-only scenarios when using a poor choice of fishery data (the non-efficient scenarios). These poor data were identified by their negative *E* and *E_1_*. We learnt that using low production sectors of fishery activity is inefficient in minimizing the cost of a habitat-based design although they are widely distributed throughout the area (Figure 5).

From Tables 4 and 5, we infer that results achieved with one habitat map are not immediately transposable to another habitat map. Using one fishery cost does not have the same effect depending on the map, and even the ranking in terms of effectiveness can be different. We also infer that each cost layer is more effective when it is used to reduce the corresponding catch loss. For instance, to minimize the *Koné* production locked in the conservation network, it is more efficient to optimize the network using the *Koné* production/cost data, and not any other cost layer. Thus, when looking at a given marine area, local and specific information is important to avoid unnecessary catch loss for conservation reasons. It is obviously possible to minimize the opportunity cost of a design by using any of the available production layers, but this is not the most effective way, and it can even be counter-productive. This conclusion also suggests that crude catch proxies should be used with caution, as they are likely to be a poor representation of the actual catch level and distribution if they are crudely mapped. Weeks et al. [13] have stressed, with proxies, the importance of using the most accurate and fine-scale possible data, but our results also suggest that most proxies, or generalization from one site to another, could lead to costly conservation mistakes [32].

We conclude here that local data are recommended. Next, a manager could ask if individual reserves need to be designed more locally with local production data and then scaled up together; or whether considerations of overall network habitat representation is more important? In other words, where is the limit, or trade-off, in terms of spatial domains? This is a study in itself that warrant further investigations. The results and recommendations will likely be dependent on reef and habitat structures, differences of livelihood between coastal communities and type of fishing practices. Here, we found that local data are needed, but this also likely reflects the Oundjo, Koné and Pouembout socio-economic differences and habitat configurations in the survey area.

Although our results confirm that habitat-driven plans would be useful for discussing the possible location of fishery restrictions and marine reserves in particular with stakeholders, conservation plans would certainly need to be revised to minimize the impacts on fishers. *Total Catches* opportunity costs of a habitat-only plan can be decreased by up to 55–61% depending on the type of habitat maps if criteria on *Total Catches* are accounted for. Although effectiveness will certainly vary in a different location than the VKP site, *Total Catches* is the most robust criteria to minimize costs as it is the highest and most widely distributed of all types of fish catches. Using a data set more easily compiled for a specific production or type of fishermen activities, would also decrease the cost of habitat-based only conservation, but far less effectively than using *Total Catches*. Here, the benefits dropped to a range of 15–25%, instead of 55–61% when using anything else than *Total Catch* information. On the other hand, mapping a *Total Catch* distribution can be a time-consuming costly task [15].

Here, only opportunity costs estimated from recent fishery production maps were considered. However other types of opportunity costs can affect fishers when setting a conservation plan [33]. This can include greater travelling distances, update of fishing gears and boats, time to rebuild knowledge on different areas, etc. Consequently, a manager must keep in mind that only one aspect of the equation was presently clarified, and that other costs need to be considered as well. In particular, Van de Geer et al. [33] emphasize taking into account the temporal variability of fishing effort. This is clearly an important aspect in a developing area like VKP. In VKP, the entire area can be considered fished and this will not change. However, catch per unit effort may change due to overfishing or better equipment coming with a booming economic activity and higher household incomes.

Finally, the 20% threshold is obviously one factor that could change the level of opportunity costs achieved by the habitat-only scenario, and the effectiveness of the cost-based scenarios. Decreasing the 20% threshold will decrease the opportunity costs, but it will also decrease the effectiveness of the network in preserving biodiversity and services. The sensitivity of the results presented here to this threshold warrants further investigation.

### Lessons for other sites

Generalizing the VKP findings to other Pacific Islands fisheries needs caution. The town of Koné is now a fairly large town by New Caledonia standards and compared to most rural locations. The level of fishing equipments used is also up-scaled compared to many Pacific rural areas [28], [34], [35], [36]. The configuration of Oundjo village is more representative of the level of activities and equipment found in most rural New Caledonia and elsewhere in Melanesia. Oundjo fishers also use a reef that offer a fairly typical Pacific island geomorphological zonation with a fringe of seagrass beds onshore, patch reefs in the lagoon, a wide sedimentary lagoon, and a barrier reef that is 4–9 km away from the village. When limiting the VKP analysis to the Oundjo fishing ground, *E* reaches up to 80% and effectiveness *E_1_* is overall higher than for the entire VKP area. For several fish families, effectiveness is much higher for Oundjo than VKP. This includes the *Haemulidae*, *Scaridae*, *Acanthuridae*, *Serranidae* and *Siganidae* families.

The effectiveness of different types of production (by fish families, by gear, by locations) in minimizing the *Total Catches* opportunity costs (*T*, *%T*, and *E_1_*) are thus likely site-dependent, in addition to being habitat map dependent as shown here (Figure 5). Additional analysis as performed for the entire VKP area, but only considering the effects on Oundjo or Koné catches, shows indeed different rankings among sites and between habitat maps (Table 6). It is therefore difficult to prioritize or recommend the systematic acquisition of any particular production data set. If one aims to produce a simplified fishery atlas (e.g., for at least one fish family, or one gear or one location) for the sake of minimizing data collection, the expected effectiveness *E_1_* would be around 25% at most, when using the detailed map and its typology of habitats for the 20% conservation objectives.

**Table 6:** Ranking of scenarios according to their effectiveness in minimizing Oundjo and Koné catch loss generated by the habitat-based scenario.

	Detailled map		Millennium map	
	Fishery ground		Fishery ground	
Rank (*E_1_*)	Oundjo	Koné	Oundjo	Koné
1	Total catches	Speargun	Total catches	Total catches
2	Gillnet	Total catches	Gillnet	Speargun
3	Lethrinidae	Scaridae	Lethrinidae	Handline
4	Handline	Siganidae	Handline	Scaridae
5	Acanthuridae	Pouembout	Mugilidae	Lethrinidae
6	Scaridae	Acanthuridae	Serranidae	Siganidae
7	Speargun	Lutjanidae	Acanthuridae	Acanthuridae
8	Serranidae	Handline	Scaridae	Serranidae
9	Mugilidae	Lethrinidae	Lutjanidae	Pouembout
10	Lutjanidae	Serranidae	Siganidae	Oundjo
11	Siganidae	Mugilidae	Speargun	Lutjanidae
12	Haemulidae	Gillnet	Haemulidae	Gillnet
13	Koné	Oundjo	Koné	Mugilidae
14	Pouembout	Haemulidae	Pouembout	Haemulidae

For each type of habitat maps, cost-based scenarios are ranked by decreasing *E_i_* (i.e., by decreasing effectiveness in minimizing the catch loss for Oundjo and Koné generated by a habitat-based scenario). In particular, we note that minimizing catch loss on one area (e.g., Koné) using the catch for the other area (e.g., Oundjo catch) is among the least effective approach.

One may suggest that to avoid extensive data collection, it may be possible to apply to each type of reefs and habitats a “standard” catch production values. This would entail a review on published production per habitats, either measured or modeled, which is likely to be unsuccessful beyond a few number of broadly defined habitats [14]. Assigning published mean production rates is a simple way to map potential productions. For instance, Newton et al. [29] suggested the 3–5 t.km^−2^.y^−1^ threshold as the limit of sustainable fishing rate for most reefs. Here, in New-Caledonia, we observed this level of catch rate on the most exploited areas, but much less elsewhere. Sustainability thresholds and actual production do not necessarily match and it is not recommended to apply one for another, as the opportunity costs would be over-estimated. Further, habitats as they can be recognized by remote sensing and ground-truthing do not necessarily convey a sense of quality and conditions, and therefore catch potential. How habitat conditions are taken into account may substantially change a conservation plan [8]. This aspect is difficult to tackle without local data. It is also another reason for avoiding generalization from one site to another.

The systematic lower costs when using Millennium maps can tentatively be explained by a greater flexibility in achieving less stringent habitat targets (Fig. 4). However, we lack more evidences from other case studies that would confirm that thematically richer maps always have worse effects for fishers. In fact, intuitively, habitat maps reflecting precisely targeted species' distribution would be optimal to minimize costs, and designing a habitat map to represent a number of species habitats will also likely lead to a more complex habitat map than the Millennium map. One way to investigate precisely this hypothesis would be to evaluate the exact link between mapped habitats and catch distribution, for different species. The distribution of catch for several key species may be related to some types of coarse habitats found in the Millennium typology (e.g. barrier reef forereef), while more detailed habitat maps may provide an artificially fragmented view of the habitat of the same species, and then less effective results. This warrants further investigation: how does mapping the exact habitat of fished species minimize costs for fishers in a habitat-based conservation plan? The fishery atlas that was used for this study would be again useful to answer this question.

## Conclusions

Using a comprehensive fine-scale coral reef fishery data set and different habitat maps, we could quantify how conservation costs can be minimized compared to a habitat-only conservation approach. The analysis was realistic, with a full range of scenarios that reflect the diversity of fishing practices found in a rural, but developing, area.

The main observation in resource-limited management situations is that habitat-based conservation schemes can be used to initiate discussions around a conservation project at a cost that is not necessarily prohibitive compared to scenario with cost optimization constraints. In terms of management recommendation, a habitat-only scenario would help move forward rapidly, but at the disadvantages of fishers in most cases. A thematically simple geomorphological (e.g., Millennium) map can provide rapidly a first low cost solution. Then, once fishery data and more detailed habitat maps become available, it would be possible to optimize the spatial design of the conservation plan to minimize opportunity costs while still meeting conservation objectives. However, depending on which fishery data set is used the benefits vary widely, and can even be negative when using marginal low-catch fisheries. Knowledge of the spatial distribution of all types of fishing activities and catch (i.e., *Total Catch*) is the most effective way, but this requires intensive data collection. The effort appears so intensive that very few coral reef fisheries are quantitatively characterized. Using instead for convenience a fraction of the *Total Catch* information may lead to non negligible improvement and lower costs for fishers (<25%), but the benefit may be seen as relatively poor, compared to habitat-only scenarios that can be used fairly quickly. It is difficult to generalize these recommendations to every configuration, depending on the urgency of the situation, the distribution of the human population, and the extent and complexity of the reefs. This is the first case study to tackle this issue and more configurations need to be investigated. Unfortunately, this study may remain isolated if no other fishery atlas are developed.

Finally, it is also recommended to use local fishery data, specific to the place, when optimizing a local conservation plan. This may sound obvious, but lack of local data may promote the use of data acquired elsewhere to fill gaps, which is likely to lead to non-optimal choices (Table 6), failure to maintain adequate incomes for fishers, disinterest of local communities (and managers) for conservation on the long run, and lack of compliance. Our results provide some guidance on the sensitivity to different types of opportunity costs (Tables 4, 5, 6), which can be interpreted as the range of risk that a manager takes when using one data layer or another to define costs. The risk can be immediately translated in economic statements, since the metrics used here (in particular *T, E_1_*) are ratio of biomass catch.
